# Supplementary material for: Antithetic hTERT Regulation by Androgens in Prostate Cancer Cells: hTERT Inhibition Is Mediated by the ING1 and ING2 Tumor Suppressors
Source: Cancers (Basel). 2021 Aug 10;13(16):4025. doi: 10.3390/cancers13164025 (PMC8391603; doi:10.3390/cancers13164025)
Supplement: Supplementary file 1 [file cancers-13-04025-s001.zip › cancers-1266152-supplementary.pdf]

cancers

MDPI

Supplementary material: Antithetic hTERT Regulation by Androgens in Prostate Cancer Cells: hTERT Inhibition Is Mediated by the ING1 and ING2 Tumor Suppressors

Sophie Bartsch, Kimia Mirzakhani, Laura Neubert, Alexander Stenzel, Marzieh Ehsani, Mohsen Esmaeili, Thanakorn Pungsrinont, Merve Kacal, Seyed Mohammad Mahdi Rasa, Julia Kallenbach, Divya Damadoran, Federico Ribaudo, Marc-Oliver Grimm, Francesco Neri and Aria Baniahmad

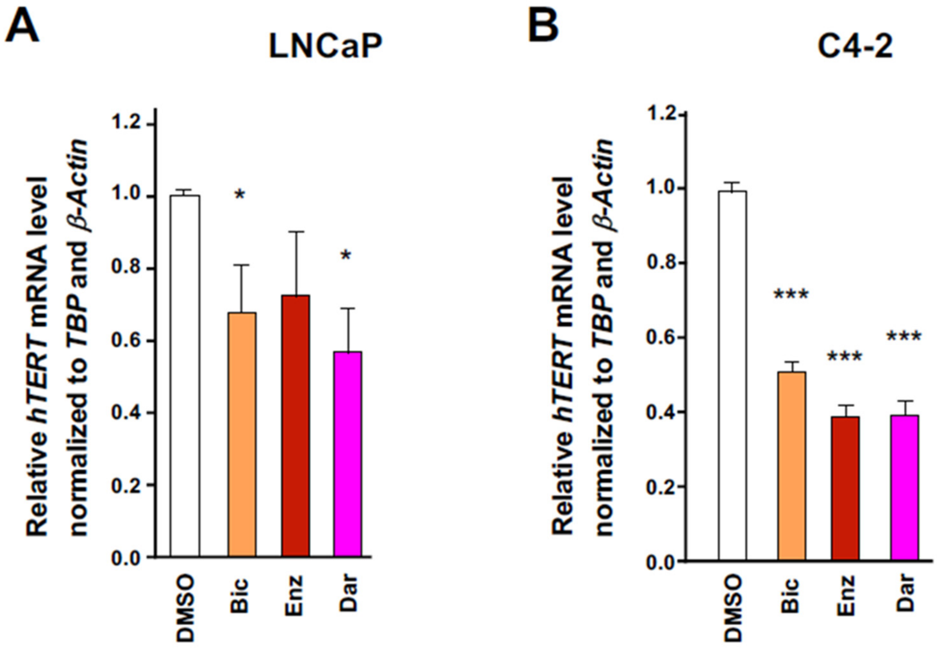

Figure S1. AR antagonists repress hTERT expression.LNCaP and C4-2 cells were treated with the established. AR antagonists bicalutamide, Bic; enzalutamide, Enz; or darolutamide, Dar (ODM-201) with each ligand at 10 µM for 72 hours in 5% FCS containing medium. DMSO served as solvent control. RNA was extracted for qRT-PCR experiments to detect changes in hTERT expression by ligand treatment. Values were normalized to both housekeeping genes TBP and b-Actin mRNAs. $n = 4$; error bars indicate, SEM, unpaired ttestwas used for statistical analysis. * $p \leq 0.05$, *** $p \leq 0.001$.

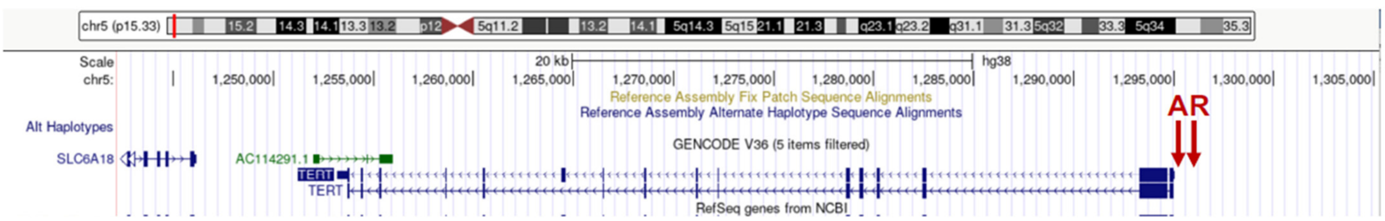

Supplemental Fig. S2: Genome Browser depicts the recruitment of AR to the hTERT promoter
Arrows indicate the androgen-dependent recruitment of AR to both the proximal and distal *hTERT* sites.

Figure S2. Genome Browser depicts the recruitment of AR to the hTERT promoter. Arrows indicate the androgen-dependent recruitment of AR to both the proximal and distal hTERT sites.

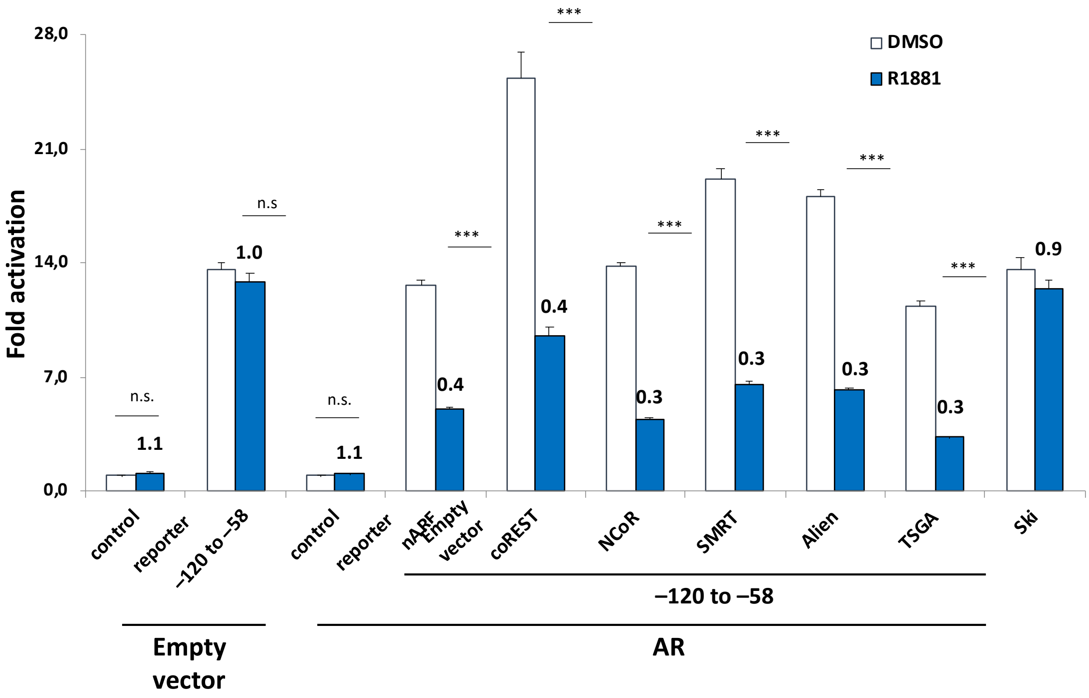

Figure S3. Overexpression of selected corepressors do not change the AR-mediated transrepression of hTERT. Reporter assays with the hTERT proximal promoter sequence −120 to −58 were performed with co-overexpression of AR and various corepressors in AR negative CV1 cells. As control empty expression vector was used. Cells were treated with either 1nM R1881 or DMSO as solvent control. The numbers above the bars indicate the Fold Repression. *** $p \leq 0.001$.

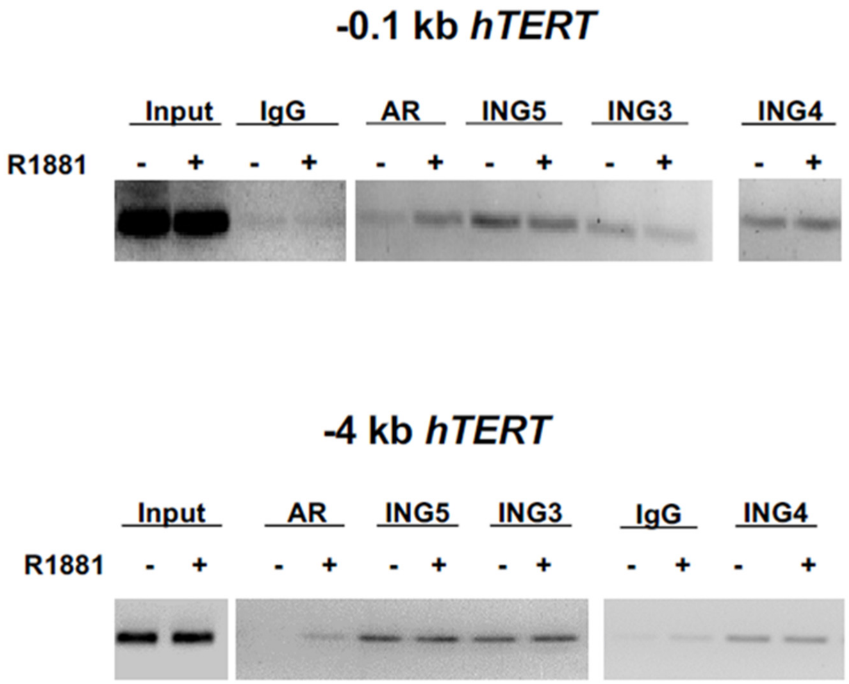

Figure S4. AR is recruited in an androgen-dependent manner to both the proximal and distal hTERT sites. ChIP-PCR was performed with antibody raised against AR and for hormone-specific control the recruitment of ING3, ING4 and ING5 were analyzed. LNCaP were treated with or without 1nM R1881. Specific primers to −0.1 kb and −4 kb regions of hTERT were used. Although ING3–5 seem to be recruited to both hTERT sites, the recruitment is androgen-independent indicating the hormone-specific recruitment of ING1 and ING2.

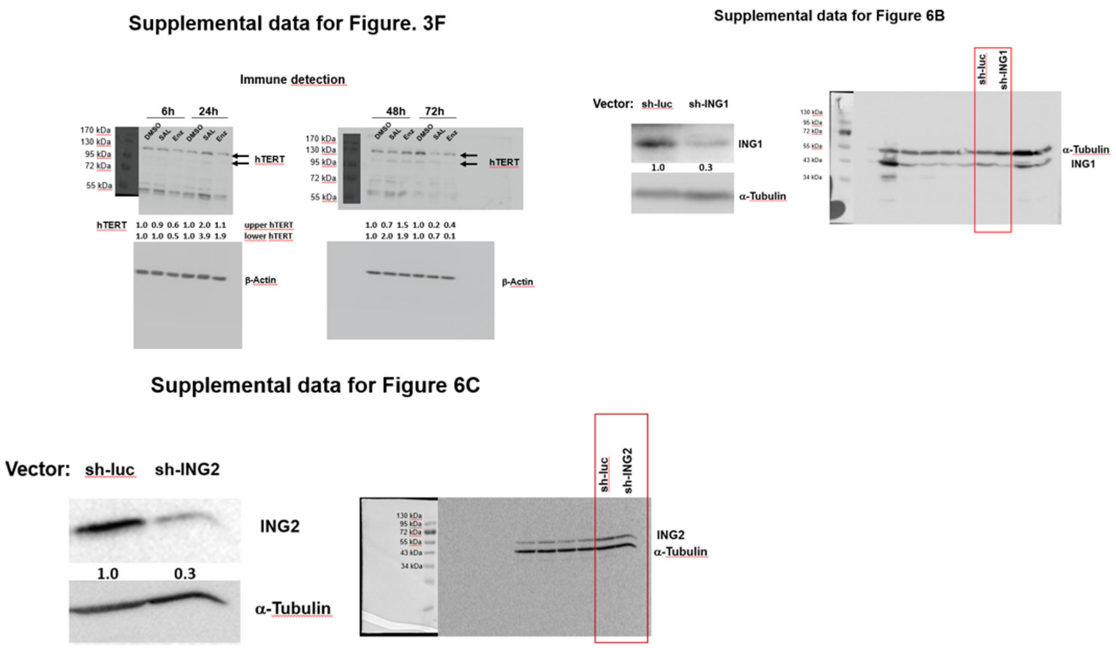

Figure S5. The Uncropped western blots.
